# Molecular epidemiology of hepatitis B virus genotypes and subgenotypes in ethnic minority populations, Yunnan province, China

**DOI:** 10.1017/S0950268821002326

**Published:** 2021-11-17

**Authors:** Zhe Dong, Jiang-Rong Li, Zhi-Xian Zhao, Lin Xu, Wen Yu, Wen-Yu Kang, Qiong-Fen Li

**Affiliations:** 1 Expanded Program on Immunization Division, Yunnan Centers for Disease Control and Prevention, Kunming, China; 2 Kunming Medical University, Kunming, China

**Keywords:** Ethnic minority, hepatitis B virus, molecular epidemiology

## Abstract

The aim of our study was to determine the distribution of hepatitis B virus (HBV) genotypes and subgenotypes in ethnic minorities in Yunnan province to provide evidence supporting the theoretical basis for hepatitis B prevention and control. We obtained serum samples and demographic data from 765 individuals reported by Yunnan province who had either acute or chronic HBV infection and were from one of 20 ethnic minority populations: Achang, Bai, Brown, Tibetan, Dai, Deang, Dulong, Hani, Hui, Jingpo, Lahu, Yi, Lisu Miao, Naxi, Nu, Pumi, Wa, Yao, or Zhuang people. We sequenced the HBV DNA and determined the genotypes and subgenotypes of the isolated HBVs. We mapped the genotype and subgenotype distribution by ethnic minority population and conducted descriptive analyses. There were four genotypes among the 20 ethnic groups: genotype B (21.3% of samples), C (76.6%), D (1.8%) and I (0.3%). The most common subgenotype was C1. There were no genotype differences by gender (*P* = 0.954) or age (*P* = 0.274), but there were differences by region (*P* < 0.001). There were differences in genotype distribution (*P* < 0.001) and subgenotype distribution (*P* = 0.011) by ethnic group. Genotype D was most prominent in Tibet and most HBV isolates were C/D recombinant viruses. The only two genotype I virus isolates were in Zhuang people. Susceptibility and geographic patterns may influence HBV prevalence in different ethnic populations, but additional research is needed for such a determination.

## Introduction

Hepatitis B is an infectious disease that is caused by the hepatitis B virus (HBV) and is a major global health threat. HBV infection can lead to serious and long-term adverse outcomes, such as liver cirrhosis (LC) and hepatocellular cancer (HCC) [1]. Hepatitis B vaccines have been available since the mid-1980s, and in recombinant form since the early 1990s. Although HBV infection has declined due to vaccination, especially among the current generation of children in China, many people living with HBV were infected before the widespread availability of the hepatitis B vaccine, during their birth. There is some horizontal transmission of HBV, mostly among adults, but the vast majority of adult infections are self-limited and do not become chronic [2]. Chronic HBV infection from perinatal infection is usually asymptomatic for decades, and complications such as cirrhosis and liver cancer emerge after the fourth decade of life. There are nearly 90 million people living with HBV in China, and as these individuals age, the prevalence of complications is increasing [3].

HBV has 10 known genotypes (A-J) and several subgenotypes [4]. Although HBV genotype or subgenotype can influence the natural history of HBV infection, antiviral treatment is not dependent on the identification of a specific genotype or subgenotype. Genotype A is associated with chronic active hepatitis; genotype D is associated with acute self-limiting hepatitis; genotype B is associated with liver cancer. Among patients with chronic HBV infection, individuals with type C and D infection have more severe clinical outcomes than those with genotype A and B infection [5].

In the United States, genotype distribution varies by geographic and demographic factors [6]. Regardless of the study site, almost all Asian patients were infected with genotypes B or C. Non-Hispanic white patients were almost all infected with genotype A or D; Black patients were mainly infected with genotype A, followed by E and D. Genotype F was the most prominent genotype of Alaska Natives [7]. Genotype G is predominant in many western countries, especially among people of Mongolian descent [8]; genotype H is frequently seen in Mexico [9]; genotypes I and J are mainly found in Asians. Correlation between race and genotype is consistent with geographical distributions in Asia, northern Europe, southern Europe and Africa. Differences in dominant HBV genotype among immigrants of various races in the United States and other countries suggest that there is a strong relationship between HBV genotype and race [10]. There may also be differences in the distribution of HBV genotypes by ethnic group. Studies in China have found differences in the distribution of HBV genotypes among ethnic groups, primarily in Xinjiang, Tibet and among other western China ethnic minorities. However, there are problems with studies on the relationship between ethnicity and HBV genotype, including inconsistent research results, insufficient sample sizes, too few ethnic groups and confounding factors.

The HBsAg carrier prevalence in southwestern China (7.9%) is significantly higher than in other regions. Yunnan province is located in southwestern China, and has the greatest geographical diversity, the highest biodiversity and the most diverse ethnic group mix in China [11]. The distribution of ethnic minorities in Yunnan is characterised by ‘scattered distribution in a large area’ and ‘aggregated distribution in a small area.’ Therefore, Yunnan's population is suitable to study epidemiology, natural history and clinical virology of HBV infection. However, to date, there has been insufficient research on HBV genotypes and subgenotypes in Yunnan [12].

## Materials and methods

### Ethical review and study population

This study was approved by the ethics committee of Yunnan CDC and carried out in accordance with national ethics regulations. The investigation plan was conducted in line with the ethical guidelines of the Declaration of Helsinki. Participants were informed of the purpose of the study, that participation was voluntary and that their data would be confidential. Participants provided informed consent before entering the study and collecting samples.

From 1 January 2017 to 31 December 2018, we enrolled 765 individuals with acute or chronic HBV infection who had been reported to the hepatitis B surveillance system from one of 16 prefectures (or cities) of Yunnan province. These individuals were from 20 different minority groups. Blood samples from included subjects met sample quality criteria. Ethnicity was self-described. For comparison with Han ethnicity, we used genotype data from 65 Han people obtained in another study that was conducted in the same region [13].

### Laboratory testing

The subjects' 765 serum samples were frozen at −20°C; repeated freezing and thawing was avoided.

#### HBV DNA preparation and amplification

In accordance with the manufacturer's instructions, we extracted HBV-DNA from 200 ml serum samples using QIA amp DNA Blood Mini Kits (Qiagen, Hilden, Germany). S-gene nested PCR amplification primers were designed consistent with protocols from the National Key Laboratory of CDC in the Chinese Center for Disease Control and Prevention and were synthesised by Shanghai Biotechnology Company. The first pair of primers was SF1:5′-cctgtattttcctgctggtggctcc-3′ and SR1:5′-gcagcaaagcccaaaagaccc-3′; the second pair of primers was SF2:5′-gttacaggcggggttttt-3′ and SR2:5′-cccatgaagttaagggagtagc-3′. Nested amplification was conducted with polymerase chain reaction (PCR) amplification kits (Takara, Japan). The first PCR amplification system consisted of Premix Tap 25 μl, 100 pmol/μl SF1 1 μl, 100 pmol/μl SR1 1 μl, sterilised double distilled water 18 μl, and HBV-DNA 5 μl, 50 μl. The second PCR amplification system consisted of Premix Tap 25 μl, 100 pmol/μl SF2 1 μl, 100 pmol/μl SR2 1 μl, sterilised double distilled water 21 μl, and first-round PCR products 2 μl, 50 μl total. PCR amplification conditions were: first, denaturation was carried out at 94°C for 5 min, followed by 35 cycles of amplification (each cycle was denaturation at 94°C for 30 s, annealing at 58°C for 40°Cs, extension at 72°C for 70 s), and extension at 72°C for 10 min. The second round of amplification was identical to the first. PCR products were eluted by 1.2% agarose gel electrophoresis. The first and second PCR products of HBV S gene fragments consisted of 931 and 646 base pairs, respectively.

#### Sequencing and genotyping of the S gene

We use the following method to determine genotype and subgenotype. After purification of the amplified S gene products, we used an ABIPrism3730X automatic sequencer (Applied BioSystems, Foster City, CA, USA) to conduct bidirectional sequencing. Sequencing results were edited by sequencer software from Gene Codes Corporation, USA. The HBV genetic reference sequences for each genotype, A-J, and subgenotype were from GenBank. Phylogenetic trees were created using the neighbour-joining method (MEGA, V5.2). The resulting HBV S gene sequences were compared with HBV genotype and subgenotype reference strains; the reliability of phylogenetic trees was tested 1000 times by bootstrap.

#### Quality control

According to the ‘Guidelines for Prevention and Treatment of Viral Hepatitis’ and quality control standards of provincial laboratories, each sample was tested twice, and only samples that were twice positive were considered HBV positive. We used positive and negative controls during DNA extraction and PCR amplification to avoid contamination.

### Statistical analysis

Baseline characteristics were described using frequencies and percentages for categorical variables and means and standard deviations for continuous variables. Pearson *χ*^2^ test was used to determine differences in categorical variables, such as the percentage of HBV genotype and gender; continuous variables, such as age, were tested for normal distribution (*P* = 0.2), with homogeneity of variance (*P* = 0.439), and then tested for differences by ANOVA. SPSS (version 16.0) was used for statistical analyses. *P* < 0.05 was considered statistically significant.

## Results

The mean age of the 765 HBV-positive subjects from ethnic minorities in Yunnan province was 37.4 ± 15.5 years; the male–female ratio was 1:1.03. We were able to amplify HBV DNA of 87.84% (672/765) and sequence 97% (649/672) of subjects' samples.

### Typing of HBV S gene

**Fig. 1.**
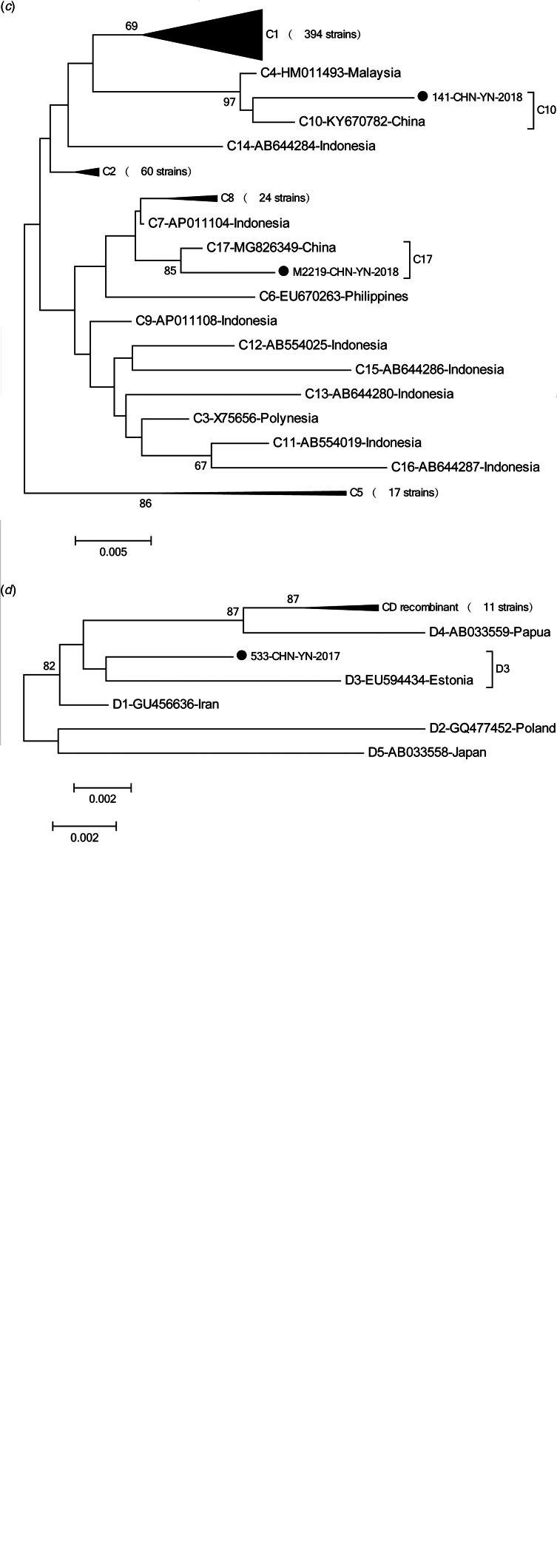
Phylogenetic analysis was based on nucleotide sequencing of the small S region. In this study, 647 strains of hepatitis B virus (HBV) were analysed and compared with the reference strains of A-J genotype and subgenotype. The accession numbers and sample numbers are shown on each tree, and before every accession number the genotype is indicated. Sequences with names that start with ‘●’ were from Yunnan. Scale bars indicate nucleotide divergence. (a) Phylogenetic tree constructed based on A-J genotype; (b) phylogenetic tree constructed based on B1-B9 genotype; (c) phylogenetic tree constructed based on C1-C17 genotype; (d) phylogenetic tree constructed based on D1-D5 genotype, including C/D recombinant.

Phylogenetic analysis of the HBV S gene (Fig. 1) showed that the 649 HBV sequenced strains were in one of four genotypes: 138 (21.3%) were genotype B, 497 (76.6%) were genotype C, 12 (1.8%) were genotype D, and 2 (0.3%) were genotype I. Among the genotype B strains, 1 was subgenotype B1, 118 were B2, 9 were B3, 7 were B4, 2 were B7 and 1 was B8. Among genotype C strains, 394 were C1, 60 were C2, 17 were C5, 24 were C8, 1 was C10 and 1 was C17. Among genotype D strains, 1 was D3 and 11 were C/D recombinant genotypes. Both genotype I strains were I1. Genotype distribution did not vary by age (*x*^2^ = 27.68, *P* = 0.274) or gender (*x*^2^ = 0.331, *P* = 0.954) (Tables 1 and 2).

**Table 1.** Gender distribution of HBV genotypes in ethnic minorities in Yunnan province

Genotype	Gender *n* (%)		Total
	Male	Female	
B	71 (51.4)	67 (48.6)	138
C	242 (48.7)	255 (51.3)	497
D	6 (50.0)	6 (50.0)	12
I	1 (50.0)	1 (50.0)	2
Total	320 (49.3)	329 (50.7)	649

**Table 2.** Age distribution of HBV genotypes in ethnic minorities in Yunnan province

Genotype	Age group *n* (%)									Total
	0~	10~	20~	30~	40~	50~	60~	70~	80~	
B	11 (8.0)	11 (8.0)	32 (23.2)	27 (19.6)	21 (15.2)	25 (18.1)	8 (5.8)	3 (2.2)	0 (0.0)	138
C	27 (5.4)	23 (4.6)	89 (17.9)	139 (28.0)	117 (23.5)	67 (13.5)	29 (5.8)	5 (1.0)	1 (0.2)	497
D	3 (25.0)	1 (8.3)	0 (0.0)	3 (25.0)	4 (33.3)	1 (8.3)	0 (0.0)	0 (0.0)	0 (0.0)	12
I	0 (0.0)	0 (0.0)	0 (0.0)	1 (50.0)	1 (50.0)	0 (0.0)	0 (0.0)	0 (0.0)	0 (0.0)	2
Total	41 (6.3)	35 (5.4)	121 (18.6)	170 (26.2)	143 (22.0)	93 (14.3)	37 (5.7)	8 (1.2)	1 (0.2)	649

The 649 sequenced HBV strains were distributed in 16 prefectures (cities) of Yunnan province, with the largest percentage being from Wenshen prefecture (20.0%), followed by Honghe prefecture (17.4%); Baoshan prefecture had the fewest strains (0.5%). Regional distributions of the four genotypes had statistically significant differences (*x*^2^ = 246.02, *P* < 0.001), as shown in Table 3.

**Table 3.** Regional distribution of HBV genotypes in ethnic minorities in Yunnan province

Prefecture (City)	Genotype *n* (%)				Total
	B	C	D	I	
Baoshan	1 (0.7)	2 (0.4)	0 (0.0)	0 (0.0)	3 (0.5)
Chuxiong	4 (2.9)	23 (4.6)	0 (0.0)	0 (0.0)	27 (4.2)
Dali	4 (2.9)	16 (3.2)	0 (0.0)	0 (0.0)	20 (3.1)
Dehong	4 (2.9)	25 (5.0)	0 (0.0)	0 (0.0)	29 (4.5)
Diqin	2 (1.4)	12 (2.4)	9 (75.0)	0 (0.0)	23 (3.5)
Honghe	29 (21.0)	84 (16.9)	0 (0.0)	0 (0.0)	113 (17.4)
Kunming	2 (1.4)	11 (2.2)	0 (0.0)	0 (0.0)	13 (2.0)
Lijiang	16 (11.6)	21 (4.2)	2 (16.7)	0 (0.0)	39 (6.0)
Lincang	9 (6.5)	29 (5.8)	0 (0.0)	0 (0.0)	38 (5.9)
Nujiang	4 (2.9)	39 (7.8)	0 (0.0)	0 (0.0)	43 (6.6)
Puer	8 (8)	80 (16.1)	0 (0.0)	0 (0.0)	88 (13.6)
Qujing	0 (0.0)	3 (0.6)	1 (8.3)	0 (0.0)	4 (0.6)
Wenshan	40 (29.0)	88 (17.7)	0 (0.0)	2 (100.0)	130 (20.0)
Xishuangbannan	11 (8.0)	35 (7.0)	0 (0.0)	0 (0.0)	46 (7.1)
Yuxi	1 (0.7)	18 (3.6)	0 (0.0)	0 (0.0)	19 (2.9)
Zhaotong	3 (2.2)	11 (2.2)	0 (0.0)	0 (0.0)	14 (2.2)
Total	138	497	12	2	649

### Distribution of HBV genotypes and subgenotypes among ethnic groups

There were statistically significant differences in the distribution of HBV genotypes (*x*^2^ = 215.8, *P* < 0.001) and subgenotypes (*x*^2^ = 625.3, *P* = 0.011) among ethnic minorities in Yunnan province. The 20 Ethnic groups included Achang, Bai, Bulang, Tibetan, Dai, De'ang, Dulong, Hani, Hui, Jingpo, Lahu, Lisu, Miao, Naxi, Nu, Pumi, Wa, Yao, Yi and Zhuang people. The distribution of HBV subgenotypes by ethnic group is shown in Table 2. HBV genetic diversity was smaller among the Han comparison group, which had genotypes B and C (Table 4). The two genotype I viruses were in Zhuang people. Tibet had a greater proportion of genotype D than other provinces, and both genotypes from Tibetan and Yi ethnic minorities were C/D recombinant (Table 5), homologous with D4. There were no statistically differences by gender (*P* = 0.129) or age (*P* = 0.439) among different ethnic groups.

**Table 4.** Distribution of HBV genotypes in ethnic minorities in Yunnan province

Ethnic group	Age ( 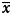 ± s)	Gender (M:F)	Genotype *n* (%)				Total
			B	C	D	I	
Achang	26 ± 5	1:2	0 (0.0)^a^	3 (100.0)^a^	0 (0.0)^a^	0 (0.0)^a^	3
Bai	36 ± 15	8:15	5 (21.7)^a^	18 (78.3)^a^	0 (0.0)^a^	0 (0.0)^a^	23
Bulang	38 ± 10	3:4	1 (14.3)^a^	6 (85.7)^a^	0 (0.0)^a^	0 (0.0)^a^	7
Tibetan	38 ± 13	6:10	0 (0.0)^a^	9 (56.3)^a^	7 (43.8)^b^	0 (0.0)^a, b^	16
Dai	38 ± 15	26:31	12 (21.1)^a^	45 (78.9)^a^	0 (0.0)^a^	0 (0.0)^a^	57
De'ang	34 ± 13	1:3	1 (25.0)^a^	3 (75.0)^a^	0 (0.0)^a^	0 (0.0)^a^	4
Dulong	25 ± 18	3:2	1 (20.0)^a^	4 (80.0)^a^	0 (0.0)^a^	0 (0.0)^a^	5
Hani	50 ± 15	55:36	25 (27.5)^a^	66 (72.5)^a^	0 (0.0)^a^	0 (0.0)^a^	91
Han	42 ± 16	49:16	53 (81.5)^a^	12 (18.5)^b^	0 (0.0)^a, b^	0 (0.0)^a, b^	65
Hui	30 ± 9	2:7	2 (22.2)^a^	7 (77.8)^a^	0 (0.0)^a^	0 (0.0)^a^	9
Jingpo	46 ± 16	4:2	2 (33.3)^a^	4 (66.7)^a^	0 (0.0)^a^	0 (0.0)^a^	6
Lahu	40 ± 16	7:2	1 (11.1)^a^	8 (88.9)^a^	0 (0.0)^a^	0 (0.0)^a^	9
Lisu	36 ± 15	13:22	2 (5.7)^a^	33 (94.3)^b^	0 (0.0)^a, b^	0 (0.0)^a, b^	35
Miao	36 ± 16	30:25	10 (18.2)^a^	45 (81.8)^a^	0 (0.0)^a^	0 (0.0)^a^	55
Naxi	34 ± 12	2:8	2 (20.0)^a^	6 (60.0)^a^	2 (20.0)^b^	0 (0.0)^a, b^	10
Nu	39 ± 10	1:2	0 (0.0)^a^	3 (100.0)^a^	0 (0.0)^a^	0 (0.0)^a^	3
Pumi	43	0:1	0 (0.0)^a^	1 (100.0)^a^	0 (0.0)^a^	0 (0.0)^a^	1
Wa	34 ± 15	16:23	8 (20.5)^a^	31 (79.5)^a^	0 (0.0)^a^	0 (0.0)^a^	39
Yao	35 ± 15	10:7	1 (5.9)^a^	16 (94.1)^a^	0 (0.0)^a^	0 (0.0)^a^	17
Yi	37 ± 17	81:86	35 (21.0)^a^	129 (77.2)^a^	3 (1.8)^a^	0 (0.0)^a^	167
Zhuang	39 ± 16	51:41	30 (32.6)^a^	60 (65.2)^a^	0 (0.0)^a^	2 (2.2)^b^	92
Total	38 ± 15	369:345	191 (26.8)	509 (71.3)	12 (1.7)	2 (0.3)	714

*Note*: each superscript letter indicates a subset of ethnic groups. At the level of *α* = 0.05, there is no significant difference between the column proportions of these groups.

**Table 5.** Distribution of HBV subgenotypes in ethnic minorities in Yunnan province

Ethnic group	Subgenotype (*n*)															Total
	B1	B2	B3	B4	B7	B8	C1	C2	C5	C8	C10	C17	CD	D3	I1	
Achang	0	0	0	0	0	0	3	0	0	0	0	0	0	0	0	3
Bai	0	5	0	0	0	0	17	1	0	0	0	0	0	0	0	23
Bulang	0	1	0	0	0	0	6	0	0	0	0	0	0	0	0	7
Tibetan	0	0	0	0	0	0	6	3	0	0	0	0	7	0	0	16
Dai	0	6	4	2	0	0	42	3	0	0	0	0	0	0	0	57
De'ang	0	1	0	0	0	0	3	0	0	0	0	0	0	0	0	4
Dulong	0	0	0	0	0	1	1	3	0	0	0	0	0	0	0	5
Hani	0	20	1	3	1	0	59	3	1	2	0	1	0	0	0	91
Hui	0	2	0	0	0	0	6	1	0	0	0	0	0	0	0	9
Jingpo	0	2	0	0	0	0	4	0	0	0	0	0	0	0	0	6
Lahu	0	1	0	0	0	0	7	0	0	1	0	0	0	0	0	9
Lisu	0	1	0	0	1	0	28	4	0	1	0	0	0	0	0	35
Miao	1	7	1	1	0	0	27	13	2	3	0	0	0	0	0	55
Naxi	0	2	0	0	0	0	6	0	0	0	0	0	1	1	0	10
Nu	0	0	0	0	0	0	2	1	0	0	0	0	0	0	0	3
Pumi	0	0	0	0	0	0	1	0	0	0	0	0	0	0	0	1
Wa	0	7	0	1	0	0	29	2	0	0	0	0	0	0	0	39
Yao	0	1	0	0	0	0	10	1	2	3	0	0	0	0	0	17
Yi	0	33	2	0	0	0	104	21	0	3	1	0	3	0	0	167
Zhuang	0	29	1	0	0	0	33	4	12	11	0	0	0	0	2	92
Total	1	118	9	7	2	1	394	60	17	24	1	1	11	1	2	649

Pairwise comparison by the ethnic group showed no statistically significant difference in genotype B. For genotype C, Han was a subset of Lisu and was statistically significantly different from other nationalities. For genotype D, Tibetan was a subset of Naxi, which was statistically significantly different from other nationalities. In genotype I, Zhuang was statistically significantly different from other nationalities.

### Comparison of HBV genotype ethnic and geographic distribution

Regardless of geographic location, the predominant HBV genotypes were consistently the same by ethnic group. As an example, the 167 Yi nationality cases (Table 6), were from 14 prefectures and cities in Yunnan. Although there were differences in genotype distribution by region (*x*^2^ = 56.63, *P* < 0.001), all viruses from Yi nationality were genotype C.

**Table 6.** Regional distribution of HBV genotypes of yi nationality in Yunnan province

Region	Genotype *n* (%)			合计
	B	C	D	
Baoshan	0 (0.0)	2 (1.6)	0 (0.0)	2
Chuxiong	4 (11.4)	17 (13.2)	0 (0.0)	21
Dali	1 (2.9)	8 (6.2)	0 (0.0)	9
Honghe	5 (14.3)	27 (20.9)	0 (0.0)	32
Kunming	1 (2.9)	7 (5.4)	0 (0.0)	8
Lijiang	14 (40.0)	16 (12.4)	2 (66.7)	32
Lincang	2 (5.7)	10 (7.8)	0 (0.0)	12
Nujiang	1 (2.9)	0 (0.0)	0 (0.0)	1
Puer	1 (2.9)	17 (13.2)	0 (0.0)	18
Qujing	0 (0.0)	1 (0.8)	1 (33.3)	2
Wenshan	4 (11.4)	8 (6.2)	0 (0.0)	12
Xishuangbanna	0 (0.0)	3 (2.3)	0 (0.0)	3
Yuxi	1 (2.9)	12 (9.3)	0 (0.0)	13
Zhaotong	1 (2.9)	1 (0.8)	0 (0.0)	2
Total	35	129	3	167

## Discussion

This study determined the HBV genotypes and subgenotypes of viruses isolated from subjects with acute or chronic HBV infection who were from any of 20 ethnic groups living in 16 prefectures of Yunnan province. Fourteen of the ethnic groups are unique to Yunnan. We found that these 20 ethnic minority populations had four HBV genotypes (B, C, D and I), most of which (76.6%) were genotype C. There were no statistically significant differences in genotype distribution by gender or age among different ethnic groups, showing that the results are likely stable, representing steady-state distributions.

Our finding that the most common genotype was C is consistent with Jing You's study [14] across all of Yunnan province. Genotype C is also the most common genotype in China (53%) [15]. Genotype C HBV inhibits the expression of HBeAg and causes immune escape, but does not affect the replication efficiency of the virus in the host, resulting in more persistent HBV infection and promoting greater deterioration of the liver. This may be the reason that some ethnic groups have greater burdens of chronic liver disease.

A large number of studies have identified the racial and geographic differences in the distribution of HBV genotypes. Some researchers have found that the distribution of HBV genotypes and the course of liver disease are closely related to ethnic background [16], and HBV infection rates differ by ethnic group in Yunnan [12]. Biological susceptibility may play a role in the difference in genotype distribution [17]. Based on our analysis of the twenty ethnic minority groups compared with the Han majority in Yunnan, we found differences in the distribution of HBV genotypes. For genotype C, Han was a subset of Lisu, which was statistically different from other nationalities, indicating that the susceptibility of Han and Lisu to genotype C was similar. Susceptibilities of Tibetan and Naxi to D genotype were also similar. Genotypes I were only seen among Zhuang people, identical to findings in 2016 [13]. Genotype I cases were found in Yunnan for the first time in China in 2011 [18], and a small number of cases have been found in recent years that may be caused by foreign carriers. However, all instances were among Zhuang people. Jinhua Hu [19] found that HBV seen in Zhuang nationality in Guangxi is mostly genotype C. Whether Zhuang nationality is more susceptible to genotype I needs further study.

HBV among Han nationality in our study was mainly genotype B, which is consistent with Hu Jinhua's research. HBV in Tibetan and Yi populations are mainly genotype C, which is consistent with what is seen in Sichuan province [20]. The distribution of C/D recombinant HBV was seen most prominently in Tibet, consistent with the results of Bin Zhou's study [21]. Most of the HBV-positive samples were from the Diqing area, which may be due to a regional prevalence of HBV genotype D in Diqing.

By comparing ethnic and geographic differences of HBV distribution, we found the uniqueness of ethnic differences. For example, Yi HBV cases were from different regions in Yunnan, but the dominant genotypes were the same, as was the case with Dai and Hani minority groups. Genetic studies have found that DNMT3B gene polymorphism varies by race, nationality, or geographic region [22]. The frequency of chronic HBV infection in the Chinese population is more than that in European populations, which may be related to NF-kB gene polymorphism [23]. In general, genetic factors can affect the incidence and prognosis of hepatitis B. Thus, the differences in HBV genotypes among different minorities may be due to human genetic differences.

Although we found ethnic differences in the distribution of HBV genotypes and subgenotypes in Yunnan, the majority of genotypes were C. This is similar to the five ethnic groups in Xinjiang (also C majority) [24] and is consistent with the dominant genotype of the entire province. However, other studies have had different findings. For example, HBV in the Bai nationality in Yunnan is mainly genotype C, while HBV in Bai nationality in Dali is mainly genotype B [25]. HBV in Han nationality in Yunnan is mainly genotype B, while the Han in Xinjiang has mainly genotype C HBV [20]. Previously, it was reported that the C/D recombinant is unique to Tibetans, while we found that Naxi and Yi were also infected with the recombinant virus. This likely indicates that in the continuous development of society, interactions between ethnic groups and regions are increasingly close, leading to the constant change in the epidemiology of HBV infection. The heterogeneity of HBV in the United States is primarily influenced by immigration [26]. This study is primarily descriptive and exploratory. Further study is needed to identify genetic differences or key social factors, such as resource inequality, to more deeply understand reasons for differences in disease burden and find a new breakthrough for hepatitis B prevention and treatment.

This study had strengths and limitations. Strengths include that the success rate of HBV DNA amplification was 87.84%, and the S gene sequencing was able to be completed in 649 cases, with a success rate of 97%. The serum quality was high and the quality control was effective. Our sample size was much larger than in previous similar studies [12], allowing a finer grain analysis of ethnic differences of HBV genotypes. Limitations include that new generation DNA sequencing technology was not used, making it impossible to obtain more information from the samples. We did not have access to special software (e.g., Simplot) to further determine HBV C/D recombinant isolates. This study had only one subject of Pumi nationality whose HBV was successfully typed, limiting representativeness for Pumi people. We plan future research to understand the distribution of HBV genotypes and subgenotypes in ethnic groups in Yunnan province using faster methods with higher sensitivities and specificities that will provide evidence, reference and insight into the prevention and control of hepatitis B.

## Conclusions

In summary, this study found ethnic differences in HBV genotype distribution along with special distributions of HBV genotypes in some ethnic groups. Some of the results differ from the results of other researchers. Importantly, the epidemiological characteristics of HBV have changed with societal changes, and there are similarities and differences in the geographic and ethnic distributions of HBV. Prospective systematic research with strategic study design and sampling methods can improve our understanding of HBV evolution. Key factors affecting the prognosis of hepatitis B infection, such as genetic, geographic, or health resource allocation, should be evaluated. Scientific study can help reduce ethnic differences in the burden of hepatitis B and the prevalence of HBV genotype C in Yunnan province, leading to an overall reduction of the burden of chronic hepatitis B infection and its complications.

## Data Availability

The data that support the findings of this study are available from Email: 1286320951@qq.com. Restrictions apply to the availability of these data, which were used under licence for this study. Data are available with the permission of the author.

## Supplementary material

For supplementary material accompanying this paper visit http://doi.org/10.1017/S0950268821002326. click here to view supplementary material
